# Utility of Consumer-Grade Wearable Devices for Inferring Physical and Mental Health Outcomes in Severe Mental Illness: Systematic Review

**DOI:** 10.2196/65143

**Published:** 2025-01-07

**Authors:** Lamiece Hassan, Alyssa Milton, Chelsea Sawyer, Alexander J Casson, John Torous, Alan Davies, Bernalyn Ruiz-Yu, Joseph Firth

**Affiliations:** 1 School for Health Sciences University of Manchester Manchester United Kingdom; 2 Central Clinical School Faculty of Medicine and Health University of Sydney Sydney Australia; 3 Centre of Excellence for Children and Families Over the Life Course Australian Research Council Sydney Australia; 4 Department of Electrical and Electronic Engineering School of Engineering University of Manchester Manchester United Kingdom; 5 Beth Israel Deaconess Medical Center Harvard Medical School Boston, MA United States; 6 Boston Children’s Hospital Harvard Medical School Boston, MA United States

**Keywords:** wearable, mental health, severe mental illness, SMI, schizophrenia, psychosis, bipolar disorder, digital phenotyping, physical health, remote monitoring, mobile health, telehealth, sleep, psychiatry, smartphone

## Abstract

**Background:**

Digital wearable devices, worn on or close to the body, have potential for passively detecting mental and physical health symptoms among people with severe mental illness (SMI); however, the roles of consumer-grade devices are not well understood.

**Objective:**

This study aims to examine the utility of data from consumer-grade, digital, wearable devices (including smartphones or wrist-worn devices) for remotely monitoring or predicting changes in mental or physical health among adults with schizophrenia or bipolar disorder. Studies were included that passively collected physiological data (including sleep duration, heart rate, sleep and wake patterns, or physical activity) for at least 3 days. Research-grade actigraphy methods and physically obtrusive devices were excluded.

**Methods:**

We conducted a systematic review of the following databases: Cochrane Central Register of Controlled Trials, Technology Assessment, AMED (Allied and Complementary Medicine), APA PsycINFO, Embase, MEDLINE(R), and IEEE XPlore. Searches were completed in May 2024. Results were synthesized narratively due to study heterogeneity and divided into the following phenotypes: physical activity, sleep and circadian rhythm, and heart rate.

**Results:**

Overall, 23 studies were included that reported data from 12 distinct studies, mostly using smartphones and centered on relapse prevention. Only 1 study explicitly aimed to address physical health outcomes among people with SMI. In total, data were included from over 500 participants with SMI, predominantly from high-income countries. Most commonly, papers presented physical activity data (n=18), followed by sleep and circadian rhythm data (n=14) and heart rate data (n=6). The use of smartwatches to support data collection were reported by 8 papers; the rest used only smartphones. There was some evidence that lower levels of activity, higher heart rates, and later and irregular sleep onset times were associated with psychiatric diagnoses or poorer symptoms. However, heterogeneity in devices, measures, sampling and statistical approaches complicated interpretation.

**Conclusions:**

Consumer-grade wearables show the ability to passively detect digital markers indicative of psychiatric symptoms or mental health status among people with SMI, but few are currently using these to address physical health inequalities. The digital phenotyping field in psychiatry would benefit from moving toward agreed standards regarding data descriptions and outcome measures and ensuring that valuable temporal data provided by wearables are fully exploited.

**Trial Registration:**

PROSPERO CRD42022382267; https://www.crd.york.ac.uk/prospero/display_record.php?RecordID=382267

## Introduction

Psychiatry, paralleling other health disciplines, has seen a rising interest in “digital phenotyping,” the concept of leveraging streams of passively collected data captured by personal digital sensors and devices to infer, monitor, and predict different states of health and illness [1,2]. A seminal UK review identified “wearables”—that is, electronic sensing devices worn on or close to the body (eg, smartphones, watches, bracelets, or garments)—as a pivotal growth area set to transform 80% of patient care pathways by 2040 [3]. The momentum driving such developments has been fueled by a number of technological, scientific, and social factors including advances in mobile sensing technology, powerful machine learning algorithms, and the broad use of smartphones among the general population, including among those with severe mental illness (SMI) [4].

Within psychiatry, the notion of using wearables to predict and ultimately prevent relapse episodes, which are both emotionally and financially burdensome, has gained traction around the world [5]. Theoretically, low-cost personal devices could detect early signs of deteriorating mental state outside of the clinic via continuously monitoring of behaviors, such as activity, travel, conversation, and human-computer interaction, in a “passive” manner [1]. Such methods are distinguishable from digital interventions that rely on “active” user input such as periodic self-reporting of symptoms using ecological momentary assessment (EMA) methods. For example, increases in psychomotor agitation and social activity that are characteristic of oncoming manic episodes in bipolar disorder could theoretically be detected using data from accelerometers, GPS, and Bluetooth encounters [6], all of which could be collected without the need for active user input. Promising results from initial exploratory, qualitative, and feasibility studies [7-9] are spurring on substantial projects aimed at relapse prevention through the use of smartphones alone or supported by other wearable devices [10,11].

Meanwhile, in other chronic diseases [12-14] and in the general population [15], monitoring and promoting physical health appears to be a far more common use case for wearables. People with SMI have substantial, life-shortening physical health inequalities [16], yet physical health care has historically been under-resourced and overlooked in such populations [17]. Increasingly, a strong case is being made to introduce digital technologies as a low-cost, scalable way to monitor and improve physical health among people with SMI [16,18]. Ensuring people with SMI have access to new technologies and are not left behind in the “digital divide” is crucial to address health inequalities. Currently, however, it is not clear what studies have used wearables in particular to monitor physical, as well as mental, health in psychiatric settings.

Evidence regarding using wearables with non-SMI populations for physical health promotion may not necessarily be generalizable on multiple grounds, including utility, feasibility, and even safety. Default activity goals suitable for other populations may be unsuitable or unattainable for people with SMI, who often have lower rates of physical activity, increased rates of sedentary behavior, and higher rates of comorbid physical conditions [16]. Thus, as with other digital health behavior interventions, wearables may require tailoring to the particular needs of people with SMI [19]. Furthermore, there is a need to understand how remote monitoring itself may interact with mental health symptoms, whether positively or negatively. For example, concerns have been raised that remote monitoring may risk inducing distress or paranoia in people with schizophrenia [20,21].

Relatively recently, narrative, scoping, and systematic reviews (eg, [22-26]) have usefully examined findings from studies using wearables among people with mental illness. Of course, this is a fast-moving area that requires regular updating. Moreover, appreciation for the nuances between these reviews is important; indeed, on closer attention, there are numerous differences in review methods, device eligibility, outcomes, and populations studied, which carry implications for future research and clinical practice. In particular, we note there has been less attention toward reviewing physical health outcomes monitored by wearables among people with SMI, leaving a distinct knowledge gap. Furthermore, acknowledging the importance of scalability and sustained participant engagement has directed the focus of this review toward consumer-grade smartphones and wearable devices in individuals with SMI, for whom the roles of these widely used devices are not well understood.

Therefore, we aimed to conduct a systematic review to examine the utility of passively collected physiological data from *consumer-grade*, digital, wearable devices for remotely monitoring or predicting changes in *mental* or *physical health* among individuals with SMI, specifically schizophrenia or bipolar disorder. To the best of our knowledge, this is the first systematic review to focus on this specific combination of criteria, allowing us to explore the area in sufficient depth to generate useful insights. We explored the types of devices used, the varieties and combinations of data passively gathered, and the associations detected between different types of passively collected data and both mental and physical health outcomes in people with SMI. Thus, the findings will be useful to inform both future research and clinical initiatives aimed at implementing wearable technologies into mental health care.

## Methods

### Overview

This review was registered on the online review protocol database, PROSPERO (CRD42022382267). It was guided by the PRISMA (Preferred Reporting Items for Systematic Reviews and Meta-Analyses) 2020 statement for reporting systematic reviews [27]; a completed checklist is available (Multimedia Appendix 1).

### Search Strategy

The searches were performed in May 2024 using the following databases: Cochrane Central Register of Controlled Trials, Technology Assessment, AMED (Allied and Complementary Medicine), APA PsycINFO, Embase, MEDLINE(R), IEEE XPlore.

The following combination of keyword search terms relevant to wearable technologies, mental illness, and health outcomes were used to find relevant papers: [psychosis OR psychoses OR psychotic OR schizophr* OR severe mental OR serious mental OR bipolar] AND [wearabl* OR smartphone OR smartwatch OR smart watch OR garmin OR fitbit OR apple watch OR withings OR oura OR passiv* collect* OR passive data OR device collected OR sensing data OR sensor OR sensors] AND [remote* OR relapse OR monitor* OR predict* OR machine learning].

The reference lists of all included papers were searched for additional studies. Only English language research papers were included in the review.

### Eligibility Criteria

The eligibility criteria for this review are described in Table 1. Briefly, studies were deemed eligible for inclusion if they collected quantitative, passively collected data about at least one physiological measure of interest (eg, sleep, heart rate, or physical activity) using a commercially available, consumer-grade wearable or smartphone device from people with SMI and related the data to a health outcome.

**Table 1:** Eligibility criteria.

Domain	Criteria
Participants	Adults (mean age 18 years and over) with SMI^a^: at least 50% with a diagnosis of psychosis, schizophrenia, or bipolar disorder (as diagnosed using any recognized diagnostic criteria) OR possible to separate people with these diagnoses from the wider sample
Design	Observational studies, randomized controlled trials (RCTs), pilot studies, and feasibility studies, provided the study collected passive data using a consumer-grade, commercially available digital wearable device (including smartphones, watches, rings, or bracelets) in a relevant population for at least 3 days; studies involving invasive or obtrusive devices (eg, glasses, helmets) and studies using only research-grade actigraphy methods were excluded.
Measures and outcomes	Studies collected quantitative, passively collected data about at least one physiological measure of interest (sleep duration, heart rate, sedentary behavior, sleep and wake patterns, steps, physical activity) AND reported associations with at least one other measure of physical or mental health not measured by the device (eg, diagnosis, psychiatric symptoms, BMI).

^a^SMI: severe mental illness.

Consumer-grade devices were defined as products designed and marketed to the general public for personal use. Studies using devices designed for medical or research purposes without a commercial use (eg, actigraphy studies) or those using invasive or obtrusive devices (eg, glasses, helmets) were ineligible. We excluded studies that only collected data requiring “active” user input (eg, EMA methods, logging step counts manually). Studies that used wearable devices as a fitness intervention but did not report analyses of passively collected data were not eligible (eg, [18]). Studies that reported results on multivariate models without specific results pertaining to features of interest to this study were not included. Studies using only GPS as a measure of activity, without other physiological metrics that were capable of distinguishing physically active modes of travel (eg, walking versus being in a vehicle), were also excluded. Relationships between geolocation data and SMI symptoms have been effectively reviewed elsewhere [28]. Studies were only included if they reported relationships between passive data and another different health (physical or mental) outcome. Thus, we excluded purely methodological studies aimed at validating passively collected measures (eg, passively sensed sleep against gold standard polysomnography).

### Study Selection Process

Titles, abstracts, authors, source, and date were imported for review into Covidence, where duplicate entries were removed prior to screening. All records were then initially screened by the lead author (LH); 15.2% (490/3229) of these records were selected on the basis of the final digit of their study import number in Covidence then independently reviewed by a second reviewer (AM). Inter-rater agreement at this stage was 98%. Reasons for exclusion at the title and abstract stage were not collected. Subsequently, at least 2 reviewers (LH plus AM or BR) independently reviewed the full texts of all short-listed papers. Inter-rater agreement on which studies to include at the full-text stage was 93%. Following full-text review, the lead author reviewed the reference lists of included papers for additional papers; the full texts of any papers she short-listed were referred for review by at least 1 other reviewer before reaching a decision about inclusion. Disagreements between reviewers regarding papers to include or exclude or reasons for exclusion were resolved via discussion.

### Data Extraction and Synthesis

Data were extracted into a purposely designed proforma in Excel and included study setting (year, country, setting, design, total duration of study, and follow-up frequency), participant demographics (diagnosis, gender, inpatient or outpatient status) and baseline characteristics (total number, number of each group, mean age, diagnostic criteria, gender, ethnicity), device details (device make and model or app), measures of activity (measures, time points collected), measures of mental and physical health (measures, time points collected), and eligible study outcomes. Due to the longitudinal nature of many of the studies, outcomes at all time points (eg, interim and on study completion) were considered eligible. Data pertaining to methods and outcomes were independently extracted by 2 reviewers (LH and AM), with any discrepancies resolved via discussion.

Meta-analysis was not appropriate due to the design of the studies and the heterogeneity of primary outcome measures reported. Outcomes were, therefore, synthesized narratively using commonly reported phenotypes (physical activity, heart rate, and sleep and circadian rhythm) to group results. Key results (for example, statistically significant associations or influential predictors included in final models) associated with mental health outcomes were also extracted and tabulated—in detail initially then summarized for clarity—organized using the aforementioned phenotypes. There were too few results to tabulate results for physical health outcomes.

## Results

### Overview

**Figure 1:**
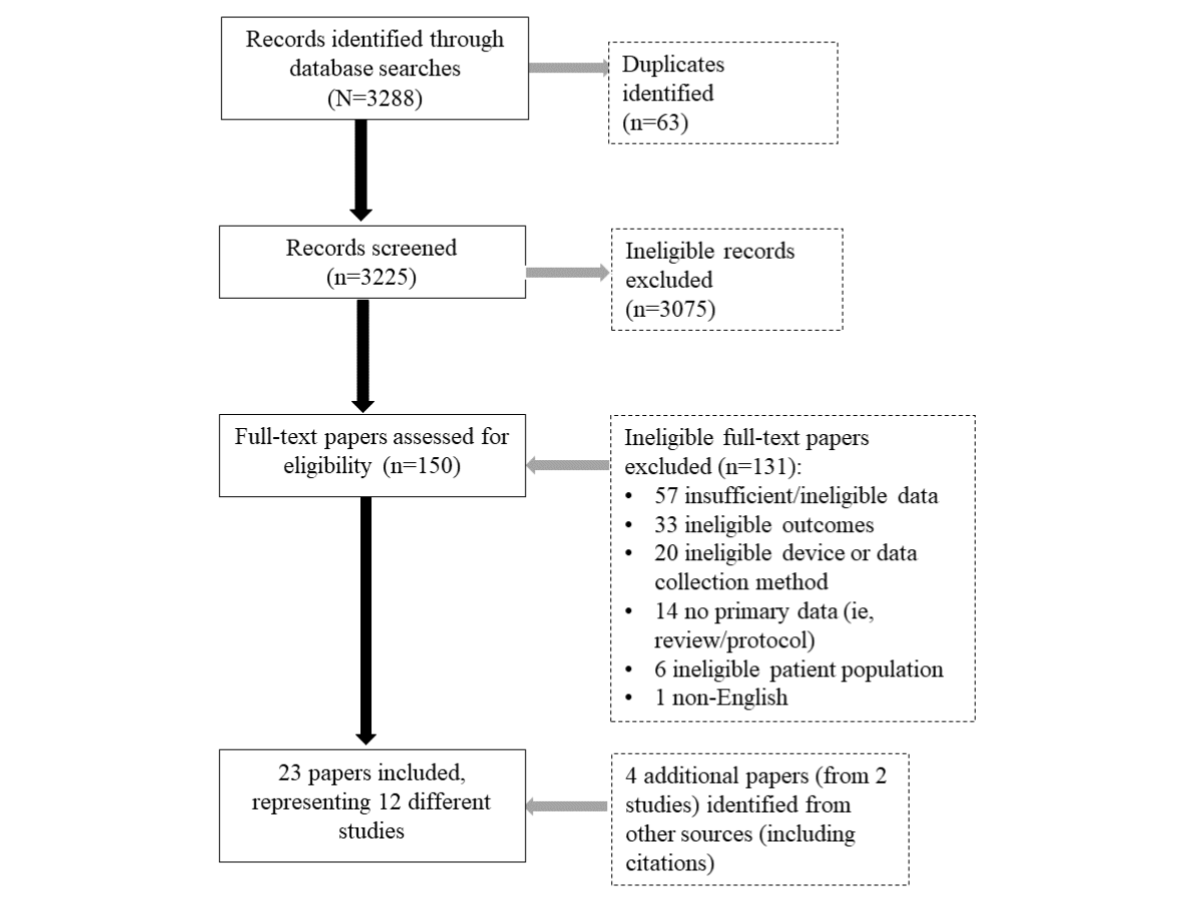
PRISMA (Preferred Reporting Items for Systematic Reviews and Meta-Analyses) flow diagram.

Overall, 3292 results were identified via database searches. Figure 1 summarizes the study selection process. We removed 63 duplicates, leaving 3229 records for screening. Following title and abstract screening, 150 full texts were reviewed for eligibility against the inclusion criteria. After this process, 131 papers were excluded. Additional searches of reference lists yielded 4 additional articles. The final number of papers included in the review was 23.

Tables 2 and 3 describe the design and characteristics of the 23 selected papers, and Table 4 indicates which phenotypes were addressed by each paper, divided into physical activity, sleep and circadian rhythm, and heart rate. Table 5 summarizes the key results of the selected studies for studies reporting mental health outcomes, divided by phenotypes.

**Table 2:** Characteristics of included papers, including study name, aim, and location.

Study name and authors (year of publication)		Key aim	Location
**Bipolar Illness Onset (BIO) study**			
	Melbye et al (2021) [29]	Investigated associations between automatically generated smartphone data and symptoms	Copenhagen, Denmark
	Stanislaus et al (2020) [30,31]	Investigated associations between automatically generated smartphone data, diagnosis, and validated measures of (1) sleep [30] and (2) symptoms, activity, and functioning [31]	Copenhagen, Denmark
**CrossCheck**			
	Buck et al (2019) [32]	Identified passively sensed indicators of persecutory ideation	New York, NY, US
	He-Yueya et al (2020) [33]	Examined how behavioral stability features can be quantified and associations with symptoms	New York, NY, US
	Lamichhane et al (2023) [34]	Used machine learning methods to predict relapse using passively sensed smartphone data	New York, NY, US
	Wang et al (2016 [35], 2017 [36], 2020 [37])	Examined associations between passively sensed smartphone data and (1) self-reported symptoms [35], (2) total BPRS^a^ scores [36], and (3) relapse [37]	New York, NY, US
**e-Prevention**			
	Efthymiou et al (2023) [38]	Examined associations between passive data and changes in relapse states	Athens, Greece
	Fekas et al (2023) [39]	Used survival analysis and machine learning models to predict risk of relapse	Athens, Greece
	Kalisperakis et al (2023) [40]	Examined associations between digital phenotypes and changes in psychopathology	Athens, Greece
	Zlatintsi et al (2022) [41]	Compared physiological and behavioral biomarkers between people with SMI^b^ and healthy controls	Athens, Greece
**Other**			
	Beiwinkel et al (2016) [42]	Examined relationships between symptoms, smartphone sensor data, and communication patterns	Lower Saxony, Germany
	Cho et al (2019) [43]	Developed machine learning models to predict mood states using passively collected data	Seoul, Republic of Korea
	Cohen et al (2023) [44]	Explored whether passive digital data could predict symptom change	Boston, MA, US; Bangalore and Bhopal, India
	Henson et al (2020 [45], 2021 [46])	Used passively collected data to (1) explore social rhythm [45] and (2) compare anomaly rates and predictors of anomalies between patients and controls [46]	Boston, MA, US
	Lahti et al (2021) [47]	Examined associations between device metrics and symptoms	Alabama, US
	Martanto et al (2021) [48]	Examined associations between digital markers and symptoms	Singapore, Singapore
	Osmani (2015) [49]	Investigated relationships between smartphone-sensed data and mood states	Tyrol, Austria
	Shin et al (2016) [50]	Assessed the association between the severity of psychopathology and physical activity	Bugok, Republic of Korea
	Song et al (2024) [51]	Examined causal relationships between circadian and sleep phase disturbances and mood symptoms	Daejeon, Republic of Korea

^a^BPRS: Brief Psychiatric Rating Scale.

^b^SMI: severe mental illness.

**Table 3:** Characteristics of included papers, including participants, duration, devices, and key variables.

Study authors (year of publication)	Sample size and diagnoses^a^	Follow-up duration	Devices	Passive data	Outcome measures
Beiwinkel et al (2016) [42]	13 with BD^b^	12 months	Smartphone (Android)	Device activity, % of day	YMRS^c^, HAMD^d^
Buck et al (2019) [32]	62 with SZ^e^	12 months	Smartphone (Android)	Time (hours) sitting still, time (hours) moving on foot	Persecutory ideation (EMA^f^)
Cho et al (2019) [43]	37 with BD and 18 with MDD^g^	2 years	Fitbit Charge HR	Daytime and bedtime steps; heart rate rhythm; sleep onset time, wake time, duration, and quality	Mood states, episodes
Cohen et al (2023) [44]	76 with SZ and 56 healthy controls	Interim analysis: mean 156 (SD 65) days	Smartphone (Android or iPhone)	Change points in sleep duration	PANSS^h^, PHQ-9^i^, GAD-7^j^, SF-36^k^, SFS^l^, PSQI^m^, WSS^n^, BASIS-24^o^, BACS^p^
Efthymiou et al (2023) [38]	19 with SZ and 10 with BD	Up to 2.5 years (mean 737 days)	Samsung Gear 3 + smartphone (Android)	Heart rate, HRV^q^, sleep duration (hours), acceleration	Relapse
Fekas et al (2023) [39]	38 with SZ or BD and 26 healthy controls	Not clear	Samsung Gear 3 + smartphone (Android)	Heart rate, acceleration, gyroscope	Relapse
Henson et al (2020 [45], 2021 [46])	45 with SZ and 43 healthy controls [45]; 63 with SZ and 27 healthy controls [46]	3 months (interim analysis) [45]; 3-12 months (mean 126 days) [46]	Smartphone (own)	Circadian routine, weekend day routine [45]; monthly anomaly rates in sleep duration [46]	PHQ-9, GAD-7, self-reported mood (EMA) [45]; diagnosis, relapse (anomaly) [46]
He-Yueya et al (2020) [33]	61 with SZ	12 months	Smartphone (Android)	“Behavioral stability” in periods (minutes) spent being still, walking, cycling, and sleeping	Self-reported mood (EMA)
Kalisperakis et al (2023) [40]	20 with SZ and15 with BD	2-14 months	Samsung Gear 3 + smartphone (Android)	Monthly, per participant (mean and SD): total motor activity, heart rate averages, HRV, steps per minute, and sleep:wake ratio	PANSS
Lahti et al (2021) [47]	40 with SZ	4 months	Garmin vivofit + smartphone (Android)	Step count, walking time (seconds), sedentary time (seconds)	PANSS, BPRS^r^, CGI^s^, CDSS^t^, PSQI, YMRS
Lamichhane et al (2023) [34]	63 with SZ	12 months	Smartphone (Android)	Average hourly total activity	Relapse
Martanto et al (2021) [48]	21 with SZ	1 week (interim analysis)	Fitbit Charge 3	Mean heart rate, mean HRV, steps, number of walks (>3 min), mean walk steps, mean walk duration (min), total duration (hours) of all sleep (light, deep, REM, total) events, mean sleep efficiency	PANSS, BNSS, CGI-S^u^, CDSS, SOFAS^v^
Melbye et al (2021) [29]	40 with BD and 21 healthy controls	2 years	Smartphone (Android)	Step counts	HAMD, YMRS, IPAQ^w^, FAST^x^, self-reported mood and activity
Osmani (2015) [49]	12 with BD	12 weeks on average	Smartphone (Android)	Physical activity score	YMRS, HAMD
Shin et al (2016) [50]	61 with SZ	1 week	Fitbit Flex	Step counts	PANSS, BMI
Song et al (2024) [51]	94 with BD and 45 with MDD	4 weeks to 4 years	Fitbit Charge HR	Sleep midpoint	Self-reported mood (EMA)
Stanislaus et al (2020) [30,31]	75 with BD, 15 relatives, and 32 healthy controls	3 years [30]; median 216 days for patients [31]	Smartphone (Android)	Sleep duration (from 12 AM to 6 AM) [30]; step counts [31]	HAMD, YMRS, PSQI [30]; HAMD, YMRS, IPAQ, FAST [31]
Wang et al (2016 [35], 2017 [36], 2020 [37])	21 with SZ [35]; 36 with SZ [36]; 61 with SZ [37]	64-254 days [35]; 2-12 months [36]; 12 months [37]	Smartphone (Android)	Sleep onset time, wake time, and sleep duration; walk and stillness durations divided into epochs (morning, afternoon, evening, and night)	Self-reported mood (EMA) [35]; BPRS [36]; relapse (including change in BPRS) [37]
Zlatintsi et al (2022) [41]	16 with SZ, 8 with BD, and 23 healthy controls	Mean of 16 months for patients	Samsung Gear 3 + smartphone (Android)	Step count, movement (short time energy), sleep:wake ratio, awake HRV, sleep HRV	Patients versus controls

^a^For brevity, in some cases, diagnoses have been pooled together into parent categories.

^b^BD: bipolar disorder.

^c^YMRS: Young Mania Rating Scale.

^d^HAMD: Hamilton Depression Rating Scale.

^e^SZ: schizophrenia.

^f^EMA: ecological momentary assessment.

^g^MDD: major depressive disorder.

^h^PANSS: Positive and Negative Syndrome Scale.

^i^PHQ-9: Patient Health Questionnaire-9.

^j^GAD-7: Generalized Anxiety Disorder-7.

^k^SF-36: Short Form Health Survey.

^l^SFS: Social Functioning Scale.

^m^PSQI: Pittsburgh Sleep Quality Index.

^n^WSS: Warning Signals Scale.

^o^BASIS-24: Behavior and Symptom Identification Scale–Revised.

^p^BACS: Brief Assessment of Cognition in Schizophrenia.

^q^HRV: heart rate variability.

^r^BPRS: Brief Psychiatric Rating Scale.

^s^CGI: Clinical Global Impressions.

^t^CDS: Calgary Depression Scale for Schizophrenia.

^u^CGI-S: Clinical Global Impressions-Severity.

^v^SOFAS: Social and Occupational Functioning Assessment Scale.

^w^IPAQ: International Physical Activity Questionnaire.

^x^FAST: Functional Assessment Staging Tool.

**Table 4:** Summary of studies using consumer-grade wearable devices to report on different passive and physiological data-based phenotypes in people with severe mental illness (SMI).

Study authors	Physical activity (n=18)	Heart rate (n=6)	Sleep and circadian rhythm (n=14)
Beiwinkel et al (2016) [42]	✓	—^a^	—
Buck et al (2019) [32]	✓	—	—
Cho et al (2020) [43]	✓	✓	✓
Cohen et al (2023) [44]	—	—	✓
Efthymiou et al (2023) [38]	✓	✓	✓
Fekas et al (2023) [39]	✓	✓	—
He-Yueya et al (2020) [33]	✓	—	✓
Henson et al (2020) [45]	—	—	✓
Henson et al (2021) [46]	—	—	✓
Kalisperakis et al (2023) [40]	✓	✓	✓
Lahti et al (2021) [47]	✓	—	—
Lamichhane et al (2023) [34]	✓	—	—
Martanto et al (2021) [48]	✓	✓	✓
Melbye et al (2021) [29]	✓	—	—
Osmani (2015) [49]	✓	—	—
Shin et al (2016) [50]	✓	—	—
Song et al (2024) [51]	—	—	✓
Stanislaus et al (2020) [30]	—	—	✓
Stanislaus et al (2020) [31]	✓	—	—
Wang et al (2016) [35]	✓	—	✓
Wang et al (2017) [36]	✓	—	✓
Wang et al (2020) [37]	✓	—	✓
Zlatintsi et al (2022) [41]	✓	✓	✓

^a^Not applicable.

**Table 5:** Summary of key results for studies reporting mental health outcomes, by diagnosis and passive data phenotype.

Passive data phenotype	Schizophrenia (SZ)	Bipolar disorder (BP)	Findings for SZ and BP
Physical activity	Higher step count: Associated with less severe symptoms [47,48,50] Achieved by healthy controls than people with SZ [41] Not associated with symptom severity [40,47] Time spent walking: In the morning associated with lower symptom severity [35] In the evening associated with relapse [37] Not associated with symptom severity [32,33,47] Changes in stillness positively associated with symptom severity [32,33,47]; stillness or sedentariness not associated with symptom severity [32,47]	Higher step count: Positively correlated with mood [29] Achieved by healthy controls than people with BD [31] Not associated with symptom severity [29,31,40] Not associated with BD in younger people [29] Timing of physical activity associated with psychiatric state [43,49]; time spent active not associated with symptom severity [42]	Larger variation in daytime movement behavior among people with SMI^a^ [41]; lower frequency or quantity and variation in movement during sleep associated with SMI [41]; total motor activity not associated with symptom severity [40]
Heart rate	Heart rate associated with symptom severity [48]; HRV^b^ associated with symptom severity [48]	Circadian rhythm of heart rate predicted mood state in bipolar disorder 1 and 2 [43]	Heart rate associated with symptom severity [40]; no association between sleep HRV (mean and variability) and symptoms [40]; increased variability in HRV features associated with SMI status [41]
Sleep and circadian rhythm	Sleep duration: Associated with various psychosis symptoms [44] Not associated with symptom severity [33,48] Later sleep onset and later waking times associated with poorer symptoms [35]; sleep efficiency not associated with symptom severity [48]; greater dysregulation in social rhythm (less routine) associated with higher symptom severity [45]	Sleep duration not associated with diagnostic status [30]; changes in sleep onset time predicted mood state [43]; causality between circadian phase disturbance (DLMO^c^) and mood symptoms in BD1 [51]	Higher mean and standard deviation of sleep:wake ratio compared with healthy controls [41]; increased mean sleep:wake ratio predicted increased negative, but not positive, symptoms [40]

^a^SMI: severe mental illness.

^b^HRV: heart rate variability.

^c^DLMO: dim light melatonin onset.

### Study Types, Participants, Devices, and Data

The 23 papers covered 12 distinct research studies reporting on observational periods ranging from 1 week to 4 years. Most of the included studies can be broadly characterized as relapse prediction studies, with the long-term goal being to use passively collected data from wearable or smartphone devices as early warning systems to detect and prevent future episodes of mental illness. We found only 1 paper with a specific focus on physical health in people with SMI, which sought to evaluate daily activity levels using a wearable wristband [50].

Separate findings from the CrossCheck study were reported in 6 papers [32-37]; CrossCheck is a smartphone-based US study deploying passive and active EMA data collection methods to identify indicators of relapse among people with schizophrenia spectrum disorders. Other studies with multiple papers included in this review included the Danish Bipolar Illness Onset (BIO) study [29-31], which recruited patients with bipolar disorder plus unaffected relatives and healthy controls, and the Greek e-Prevention project [38-41]. Only 1 paper [44] included participants from a low or middle-income country, namely India.

A variety of measures were used to measure mental health outcomes including self-report rating scales of overall mood or individual symptoms (eg, persecutory ideation), often obtained via EMA methods; validated scales administered by clinicians, for example the Positive and Negative Syndrome Scale (PANSS; [52]), Young Mania Rating Scale [53], and Hamilton Depression Rating Scale [54]; and other outcomes or composite outcomes such as relapse.

Data only on participants with schizophrenia spectrum disorders were included in 12 papers, 7 papers only included participants with bipolar disorder, and 4 e-Prevention study papers included participants from both diagnostic groups. All papers described outpatient samples, except Shin and colleagues [50] who only recruited hospital inpatients and Song and colleagues [51] who recruited both outpatients and inpatients. Study sample sizes varied between 12 and 139 participants (including controls and diagnoses other than SMI). Some studies (eg, e-Prevention, CrossCheck) included only subsets of data for participants with valid data, for example using eligible Android devices or with sufficient data quality. After accounting for overlap between samples, we estimated that over 500 participants with SMI were included across the selected studies in total (using the largest reported sample per diagnosis per study, estimated mean=49.4% female, 17 papers).

Papers predominantly reported on studies that used smartphones to support passive data collection and running a variety of study-specific (eg, CrossCheck), open-source (eg, Beiwe, MindLamp), and other apps (eg, Monsenso). With the exception of studies using open-source apps, papers were limited to reporting on patients using Android smartphones. The use of wrist-worn hardware to support passive data collection was reported in 8 papers [38,40,41,43,47,48,50,51]; the hardware specifically included the Samsung Gear 3 (used by the e-Prevention project), Garmin vivofit, and several Fitbit models (Fitbit Charge, Fitbit Charge HR, Fitbit flex).

### Physical Activity

Physical activity was the most common metric presented in the selected studies, appearing in 18 of the 23 papers (Table 4). Physical activity was measured in a variety of ways. This included, most commonly, step counts [29,30,39-41,43,47,48,50]; the duration of time spent walking, being still, or being sedentary [32,33,35-37,47,48]; and other measures of total activity, such as linear acceleration [34,38,42,49]. The use of smartwatches to support data collection was reported by 8 papers [38-41,43,47,48,50]; the rest used smartphones only.

The evidence regarding the association between physical activity and symptom severity as measured by wearable devices was inconsistent (Table 5). The review found some evidence that increased overall daily physical activity, whether measured by steps or walking time, could be associated with improved mood in bipolar disorder [29] and less severe symptoms in schizophrenia [47,48]. For example, Martanto et al [48] reported multiple negative correlations between step counts and symptom severity using PANSS subscales.

Nonsignificant associations, however, were far more frequently reported between physical activity and symptoms across all diagnoses [29,31,32,40,42,47,50]. Studies were typically exploratory, testing multiple combinations of predictors and outcomes, with varying strategies for dealing with multiple testing. For example, Wang et al [36] developed a Brief Psychiatric Rating Scale score prediction model using passive sensing data in which 434 features were originally tested; however, none of the 18 features included in the final model addressed physical activity. Even where physical activity (eg, acceleration, gyroscope) data were included in relapse prediction models, some authors noted inconsistent [39] or weaker contributions to model performance relative to other modalities [34].

The detail of some studies may shed some light on the subtleties in the nature of the relationships between physical activity and symptoms among people with SMI. In particular, some results imply that timing of activity could be important in both schizophrenia and bipolar disorder. Three studies—1 on schizophrenia [35,37] and 2 on bipolar disorder [43,49]—found significant associations when activity was divided into time of day. For example, Wang and colleagues [35,37] found that walking in the morning was negatively associated with symptoms among people with schizophrenia, while evening walking was positively associated with symptoms. Similarly, steps taken at night made stronger contributions to mood episode prediction models among people with bipolar disorder than steps taken during the day [43]. Conversely, the absence of activity—or, at least, changes in activity—may also be important in the context of schizophrenia, at least. Although between-participant analyses showed that the duration of time spent sedentary or still was not a predictor of symptoms [32,47], some analyses from the CrossCheck study suggested that changes in stillness over time (within-participants) were indeed significant predictors [32,33].

Findings in terms of other health outcomes were limited. Lahti et al [47] found that activity data (walking time, sedentary time, and steps) predicted the sleep duration component (but not global score or other items) of the Pittsburgh Sleep Quality Index [55] among people with schizophrenia. Shin et al [50] found no significant correlation between daily activity (steps) and BMI among people with schizophrenia. Both studies that looked at levels of psychosocial functioning—1 with people with bipolar disorder [31], the other among people with schizophrenia [48]—found significant associations with step count.

### Sleep and Circadian Rhythm

Measures relating to sleep or circadian rhythm were reported by 14 papers. Measures of sleep duration and sleep:wake ratio were most frequently cited, though some studies went into further detail citing metrics such as sleep efficiency, onset time, waking time, midpoint, and duration of sleep stages (eg, light, deep, and rapid eye movement [REM] sleep). The use of smartwatches to support data collection was reported by 6 papers [38,40,41,43,48,51].

No studies indicated that absolute sleep duration was significantly associated with symptom severity [30,48]; however, 1 study did find an increased sleep:wake ratio among people with SMI than for healthy controls [41]. Several papers did, however, report evidence that changes or irregularities in sleep patterns and circadian rhythm within participants over time were associated with psychiatric symptoms in both schizophrenia [44-46] and bipolar disorder [43,51].

Again, the addition of time-related data to sleep data appeared to yield some interesting associations. Wang et al [35] noted that waking up earlier was generally linked to more positive mental health states among people with schizophrenia, while going to bed later was linked with more negative states. In later papers from the CrossCheck study, however, sleep onset and offset features were less important relative to other features and were not selected in the final symptom and relapse prediction models [36,37]. A study with people with bipolar disorder found that regularity in sleep onset time ranked more highly relative to other features across various mood state prediction models, including sleep length [43]. Efthymiou et al [38] noted that adding day of the week information and temporal encoding data (the order of samples) to sleep duration data usefully contributed to psychosis relapse prediction models.

Research by Song et al [51] indicates that the subtle distinction between sleep and circadian phase disturbances (eg, light exposure patterns) may be important, at least for people with bipolar disorder. Using mathematical models and sleep midpoint data, they were able to estimate the timing of dim light melatonin onset, an important biomarker of circadian phase. They demonstrated causal relationships between circadian phase but not sleep phase, disturbances, and mood symptoms in people with type 1 bipolar disorder. Moreover, they postulated that sleep phase disturbance induced circadian phase disturbance, providing insights into causal pathways.

### Heart Rate

Of the selected papers, 6 reported on heart rate data (Table 4), all of which used smartwatches [38-41,43,48]. Heart rate data types collected included mean heart rate (while awake and asleep), cyclical measures of heart rate indicative of circadian rhythm, various measures of heart rate variability (HRV; eg, means and standard deviations while awake and asleep), and heart rate rhythms over extended periods (eg, 48 hours).

Zlatintsi et al [41] looked at differences among people with SMI compared with healthy controls and found some signs of increased heart rate variability, particularly during wakefulness. Of the e-Prevention project papers, 2 reported that heart rate measures enhanced performance of relapse models [38,39].

The remaining studies looked at associations between heart rate features and psychiatric symptom severity. Two separate studies—1 with people with schizophrenia [48] and 1 with a mixed sample of people with SMI [40]—reported significant positive associations between mean heart rate and positive symptoms using the PANSS; however, neither of these studies found any associations between mean heart rate and negative symptoms. Significant inverse relationships were also found between HRV measures (mean and standard deviation) and negative symptoms [40,48]. Inclusion of BMI in models, however, nullified associations between mean HRV and negative symptoms in 1 study [40].

Finally, Cho and colleagues [43] found that various parameters indicative of heart rate circadian rhythms differed between people with bipolar disorder with higher and lower self-reported mood scores; subsequently, the average circadian rhythm of heart rate was identified as the most influential feature in mood state prediction models in type 1 but not type 2 bipolar disorder.

## Discussion

### Principal Findings

This review examined mental and physical health outcomes associated with passively collected physiological data in people with SMI in studies using consumer-grade wearable devices. We found 23 eligible papers reporting on 12 different studies, including data from over 500 participants with SMI, predominantly from high-income countries. Most of the selected studies collected physical activity data, followed by sleep and circadian rhythm data; a few studies used smartwatches to collect heart rate data.

The focus on mental health outcomes dominated the literature reviewed. There was a distinct evidence gap in terms of using passively collected wearables data to build a picture of physical health among people with SMI and to explore relationships with physical health indicators and outcomes such as blood pressure, BMI, and cardiorespiratory fitness. This presents a “missed opportunity” so far in the field, given that such technologies are typically designed and focused on physical health metrics and it is the heightened risk of physical diseases (rather than psychiatric relapse) that accounts for the majority of premature mortality in SMI populations [16]. Indeed, most eligible papers were from longitudinal cohort studies focused on relapse prevention. Although consumer-grade wearable devices have previously been used as physical health interventions among people with SMI [56], analyses of passively collected data relevant to profiling physical health have not necessarily been published. We found just 1 eligible study that explicitly addressed physical health and tracked participants for just 1 week [50]. Although relapse prevention is unquestionably an important goal, making better use of these unobtrusive and widely used devices to improve the physical health monitoring of people with SMI has been an underexplored avenue for addressing the key health inequalities that affect this population.

This review did find some evidence of digital markers indicating relationships between patterns of physiological metrics and psychiatric states or symptoms, largely in the manner expected: Lower levels of activity, higher and more variable heart rates, lower and more variable HRV, and later and irregular sleep onset times were associated with psychiatric diagnoses or poorer symptoms in some analyses. However, the heterogeneity in methods—including sampling, devices, measures, and statistical approaches—presents a complex picture when interpreting findings. Although we can conclude, on the basis of this review, that certain physiological metrics measurable by commercial devices likely have value for monitoring health, these are not necessarily always straightforward linear relationships; more evidence is needed before we can make conclusive statements about predictive utility in the context of individual psychiatric symptoms or diagnoses.

### Implications for Future Digital Phenotyping Research

As others examining digital phenotyping more broadly have observed [22], studies used a wide variety of variables (or features) and combinations derived from different devices as predictors. One barrier to combining data sets and synthesizing findings on a greater scale may be that there is a lack of standards pertaining to research using wearable devices, including smartphones. Currently, for example, there is no consensus regarding how concepts such as “activity” should be measured in the context of digital phenotyping [31]. This means that different studies use different variables (eg, steps, walking time, or stillness) and different definitions of variables between studies. A degree of this is arguably to be expected: Different symptoms may require measurement in different conditions, and digital phenotyping in psychiatry is still in an “exploratory” phase. Nonetheless, it could preclude the types of evidence syntheses and meta-analyses required to advance the field. Other areas that may benefit from greater standardization either in study design or reporting include trial preregistration [57]; greater harmonization of data formats; and providing adequate descriptions of data, devices, and software (eg, versions and operating systems).

Nonetheless, despite the heterogeneity of studies, we were able to extract some useful general observations. We observed that some authors uncovered digital markers when incorporating more fine-grained temporal markers (eg, sleep onset time, daytime steps) into analyses [35,37]. Indeed, this would appear consistent with how symptoms can present in people with mental illnesses, such as experiencing morning fatigue or excessive nocturnal activity. It is thus possible that standard daily measures, such as daily steps or sleep duration, may be suitable for mass markets interested in wellness but overly coarse to enable the types of pattern detection crucial for research and clinical purposes. Collecting raw, unprocessed data may be optimal for retrospectively pooling data sets and creating sufficiently large sample sizes to allow novel analytical methods to be applied in future; however, this may not always be available for commercial devices. On this basis, we recommend future research use at least some form of epoch data (and other time or seasonality-related data) wherever available.

Furthermore, considering the trend for multimodal, data-driven approaches, the value of individual passively collected physiological data items may be best understood and maximized in the context of other variables—both within and between individuals. Considering patterns within individuals to understand routines and instability or anomalies in such patterns over time was a commonly tested approach among the selected studies [33,46]. This is consistent with the notion of individuals having individualized “relapse signatures” that precede relapse events [58]. Thus, future research should exploit the advantages of continuous monitoring offered by wearables and consider using methods and measures that attend to variability and not just symptom thresholds. This mirrors the findings of a recent review examining digital phenotyping in people with depression, which found associations between symptom severity and variability in sleep, affect, and psychomotor aspects of depression as captured by digital technologies [59].

We also found several papers using machine learning approaches to yield useful insights from complex, high-resolution data. Although we had to exclude some interesting machine learning papers included by other reviews [60] for lack of detail about features derived from passively collected physiological data, this general approach seems promising and an opportunity to build personalized models. Furthermore, although we focused on passively collected data in this review, actively collected, self-reported symptom data also clearly have value. Combining active and passive data collection may offer ways to combat disengagement and reduce missing data, which are well-recognized problems in digital psychiatry [61,62].

Although feasibility and acceptability outcomes were out of scope for this review, we note these are essential prerequisites for digital health interventions involving wearables. As with any digital intervention, care must be taken to ensure benefits outweigh potential harms. Study designs that give due attention to symptom monitoring and reactivity to digital methodologies may offer one way of guarding against unintended health effects. Although exacerbation of paranoia symptoms among people with schizophrenia has been one notable concern, studies of digital health interventions reporting on such outcomes have not yet found this to be a significant issue [20]. Specifically regarding the use of commercial devices, we also note potential challenges relating to data governance, privacy, and digital equity. Indeed, previous mental health data science initiatives have noted the need for transparent governance arrangements and sustained engagement of patients in research, a sentiment that we also echo [63]. We summarize our recommendations for future research using physiological data from consumer-grade wearables in Table 6.

**Table 6:** Recommendations for future research using physiological data from consumer-grade wearables in digital psychiatry.

Domain	Recommendations
Research topics	Investigate the feasibility and acceptability of physical health monitoring in SMIa using passive data from consumer-grade wearables Investigate the utility of passive data from consumer-grade wearables for physical health monitoring purposes in SMI and whether this mirrors relationships in other patient populations Investigate relationships between heart rate data from consumer-grade wearables and mental health outcomes in people with SMI
Research methods	Collect and use raw, unprocessed data wherever possible Use at least some form of epoch data wherever available Consider including measures of variability between and within individuals Consider machine learning approaches to leverage multimodal wearable data and explore the use of personalized prediction models Consider combining active and passive data collection to combat disengagement and reduce missing data Consider whether consumer-grade or research-grade devices are the most suitable design choice for the study
Standardized reporting	Include descriptions of devices, operating systems, and software versions, where available Include definitions of activity (eg, total activity, walking) and other key outcome variables Include adequate descriptions of sample characteristics including diagnosis and symptom severity Describe results by diagnosis or provide justification for pooling results
Responsible research practices	Encourage trial preregistration Address inequalities in evidence resulting from under-representation of data from low and middle-income countries Consider including measures to capture reactivity to digital methodologies Consider whether raw data could be stored and made available for future research, subject to suitable ethics and governance arrangements Involve patients and the public in research design to improve the relevance, transparency, and acceptability of research

^a^SMI: severe mental illness.

### Limitations

This review was limited to consumer-grade wearables and physiological variables; thus, we excluded some well-cited studies using actigraphy, research-grade devices, and other passively sensed device-based measures (eg, GPS, call logs) to detect symptoms and relapse in people with SMI (eg, [64]). The decision to opt for a narrower focus was based on our interest in scalability of devices in routine clinical practice (hence, examining consumer-grade devices) and physical health promotion (hence, physiological sensing), offering a novel viewpoint. Our findings should be viewed in this context, acknowledging that consumer-grade wearables may be less accurate than research-grade devices; thus, the nature of the associations observed do not represent the full picture.

The studies that were included had some methodological limitations. In addition to the heterogeneity of passive data measures used, several studies were relatively small-scale or short in duration [48-50]. High levels of attrition were common, and monitoring duration varied. The majority of participants included in passive data analyses were Android phone users, and all except 1 study [44] recruited participants from high-income countries. Thus, sampling bias is potentially an issue. Similar problems have been noted by authors of previous reviews [25,60,65], and, based on our own findings, we join their calls for addressing evidence inequalities, greater optimization of methods, and standardized reporting. Caution should be exercised when generalizing results, especially given that, in several cases, multiple papers reported outcomes from overlapping samples. Although we tried to delineate outcomes within particular diagnoses and subtypes, this was not always possible where evidence was scarce or studies pooled results for people with SMI. Finally, although a thorough examination was beyond the scope of this paper, we recognize that a lack of common definitions for key measures such as relapse can be problematic [66] and could have affected the results.

### Conclusions

This review shows that consumer-grade wearables are capable of detecting at least some digital markers indicative of psychiatric symptoms or mental health status among people with SMI, but more evidence is needed to demonstrate clinical utility in the context of particular diagnoses. Furthermore, our review identified a paucity of research examining the use of consumer-grade devices for physical health outcomes in people with SMI. Given that physical health is both the primary concern of such technologies and the leading cause of mortality in SMI, this represents a missed opportunity and further suggests this underserved population may be excluded from potential physical health improvements afforded by such devices. To advance this field, researchers should consider choice of predictors (features) and outcomes carefully, use temporal markers, and work toward agreeing on common measures to aid comparability and meta-analyses. Furthermore, those conducting future studies involving wearables should consider opportunities for physical health monitoring and promotion.

## Appendix

Multimedia Appendix 1 PRISMA (Preferred Reporting Items for Systematic Reviews and Meta-Analyses) checklist.

## Abbreviations

AMED: Allied and Complementary Medicine
HRV: heart rate variability
PANSS: Positive and Negative Syndrome Scale
PRISMA: Preferred Reporting Items for Systematic Reviews and Meta-Analyses
REM: rapid eye movement
SMI: severe mental illness
